# Inflammation during Lung Cancer Progression and Ethyl Pyruvate Treatment Observed by Pulmonary Functional Hyperpolarized ^129^Xe MRI in Mice

**DOI:** 10.1155/2021/9918702

**Published:** 2021-06-28

**Authors:** Atsuomi Kimura, Seiya Utsumi, Akihiro Shimokawa, Renya Nishimori, Neil J. Stewart, Yoshihiro Kamada, Hirohiko Imai, Hideaki Fujiwara

**Affiliations:** ^1^Department of Medical Physics and Engineering, Area of Medical Imaging Technology and Science, Division of Health Sciences, Graduate School of Medicine, Osaka University, Osaka, Japan; ^2^POLARIS Imaging Sciences, Department of Infection, Immunity & Cardiovascular Disease, University of Sheffield, Sheffield, UK; ^3^Department of Advanced Metabolic Hepatology, Graduate School of Medicine, Osaka University, Osaka, Japan; ^4^Division of Systems Informatics, Department of Systems Science, Graduate School of Informatics, Kyoto University, Kyoto, Japan

## Abstract

This study aimed to assess the suitability of hyperpolarized ^129^Xe (HPXe) MRI for noninvasive longitudinal evaluation of pulmonary function in preclinical lung cancer models. A mouse model of lung cancer (LC) was induced in 5 mice by intraperitoneal injection of urethane, while a negative-control (NC) mice (*N* = 5) was prepared by injection of saline solution. Longitudinal HPXe MRI was performed over a 5-month period to monitor lung ventilation and gas exchange. The treatment efficacy of ethyl pyruvate (EP), an anti-inflammatory drug, to the mouse LC model was monitored using HPXe MRI by commencing administration of EP pre (early-phase) and 1-month post (late-phase) injection of urethane (*N* = 5 mice for each group). Gas-exchange function in LC mice was significantly reduced at 1-month after urethane injection compared with NC mice administered with saline (*P* < 0.01). Thereafter, it remained consistently lower than that of the NC group for the full 5-month measurement period. In contrast, the ventilation function of the LC model mice was not significantly different to that of the NC mice. Histological analysis revealed alveolar epithelial hyperplasia in LC mice alveoli at 1 month after urethane injection, and adenoma was confirmed 3 months after the injection. The early- and late-phase EP interventions were found to improve HPXe MRI metrics (reduced at 1 month postinjection of urethane) and significantly inhibit tumor growth. These results suggest that HPXe MRI gas-exchange metrics can be used to quantitatively assess changes in the precancerous lesion microenvironment and to evaluate therapeutic efficacy in cancer. Thus, HPXe MRI can be utilized to noninvasively monitor pulmonary pathology during LC progression and can visualize functional changes during therapy.

## 1. Introduction

Lung cancer is the leading cause of cancer mortality worldwide [1]. In Japan alone, the mortality rate and the number of lung cancer associated deaths reached 59.5% and 74,120, respectively, in 2017, and these numbers are predicted to further increase [2]. Moreover, there is a lack of effective lung cancer treatments and thus the development of novel drugs, for example, molecular targets and immune checkpoint inhibitors [3, 4], is of significant interest. Preclinical studies using small animals such as mice and rats are key steps in the assessment of the treatment efficacy of new drugs prior to consideration for clinical trials. Magnetic resonance imaging (MRI) is safe, nonionising and well-suited to repeated, longitudinal measurements and is therefore a powerful tool for treatment response assessment in a preclinical setting.

Hyperpolarized (HP) noble gas MRI is gaining significant attention in the preclinical arena as a robust method to evaluate respiratory abnormalities in ventilation and microstructure for small animal lung imaging [5]. In particular, hyperpolarized ^129^Xe (HPXe) can be imaged at low cost using natural abundance xenon gas [6]. It dissolves in lung tissues and red blood cells and gives rise to well separated peaks reflecting the chemical environment of the dissolved medium. These properties allow the evaluation of gas-exchange as well as ventilation function of the lung [7]. To make the most of these properties for preclinical lung functional assessment, we have developed a preclinical HPXe MRI system with custom-built flow-mode polarization apparatus to produce HPXe and deliver it to a mouse in a closed loop [8]. Hitherto, we successfully applied this system to early pathological detection and evaluation of treatments in chronic obstructive pulmonary disease (COPD) and pulmonary fibrosis using mice models [9, 10].

Whilst HP noble gas MRI is well-suited to the study of the respiratory pathologies, its application to lung cancer has been constrained by the lack of interaction between noble gases and cancer cells and a relative insensitivity to metabolic information. Previous studies were performed only after solid tumor nodules had fully developed [11–18], with few studies exploring dissolved-phase ^129^Xe in lung cancer models to date [14, 17]. In light of these previous reports, we attempted to perform an exploratory HPXe MRI study in preclinical lung cancer. That is, in this work, we evaluated changes in pulmonary ventilation and gas exchange function during progression of pathology in a mouse model of lung cancer induced by urethane injection [19] using HPXe MRI. Additionally, to investigate the relationship between pulmonary functional and morphological changes during cancer progression, we performed histology measurements and compared them with the HPXe MRI metrics. Finally, we used HPXe MRI to monitor the response of pulmonary function to treatment with EP in the mouse model of lung cancer.

## 2. Materials and Methods

### 2.1. Animal Preparation

All experimental and animal care procedures conformed to the guidelines of Osaka University.

**Figure 1:**
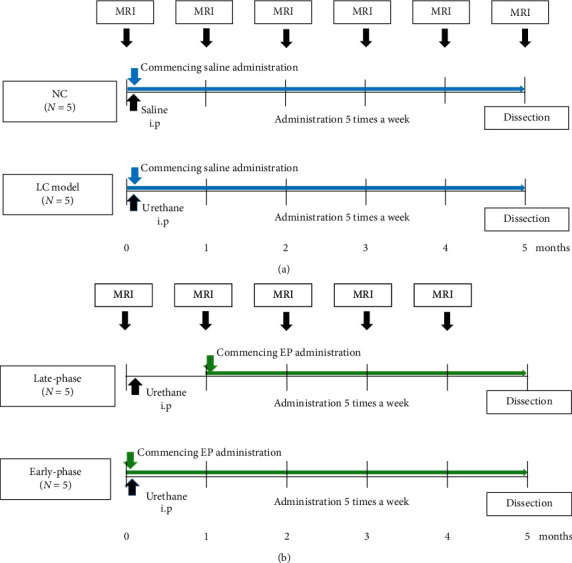
Schematic diagram of experimental design of the longitudinal measurement of (a) lung cancer progression and (b) EP treatment. Late-phase and early-phase: late- and early-phase intervention groups.

Ten male, eight-week-old, ddY mice (Japan SLC, Inc., Shizuoka, Japan) were included in this study. Mice initially underwent lung functional assessment with HPXe MRI at baseline, i.e., preceding any disease model treatment (0 months). Subsequently, mice were divided into two groups: a negative-control (NC) group (*N* = 5) and a lung cancer model group (LC group, *N* = 5). To induce lung cancer, a 500 *μ*L saline solution of urethane (Tokyo Kasei, Tokyo, Japan) was intraperitoneally administrated to each mouse of the LC group (500 mg/kg body weight). The NC mice were intraperitoneally administered with a 500 *μ*L saline solution. Subsequently, all mice were intratracheally administered with a 20 *μ*L solution of saline daily for five months to replicate the experimental procedure used for ethyl pyruvate (EP) administration detailed as follows. Lung function was assessed in the two groups by HPXe MRI at 1, 2, 3, 4, and 5 months from the initial saline or urethane administration. The schematic diagram of experimental design is shown in Figure 1(a). Throughout MRI, mouse body temperature was maintained with warm water circulating through a rubber tube placed on the abdomen. As such, HPXe MRI was performed without tracheal intubation or tracheotomy and hence was entirely noninvasive. The survival rate of the whole procedure was 100% for both groups. After 5 months, mice were euthanized with a lethal dose of carbon dioxide gas to measure the number and size of lung surface tumors.

In addition to the NC and LC groups, a third, ethyl pyruvate (EP), an anti-inflammatory drug, treated group was prepared (*N* = 10). The EP-treated group was divided into two subgroups: an early-phase intervention group (*N* = 5) and a late-phase intervention group (*N* = 5). The schematic diagram of the experimental design of the EP treatment is shown in Figure 1(b). Initially, mice in the EP-treated group underwent a HPXe MRI baseline scan (0 months). Then, for the late-phase intervention group, a saline solution of urethane was intraperitoneally administrated to each mouse in the same manner as for the LC model mice. In addition, beginning 1 month after the urethane injection, a 20 *μ*L solution of EP in saline (2.6 mg/kg, Tokyo Chemical Industry Ltd, Tokyo, Japan) was intratracheally administered to each mouse on five consecutive days within one week for four months. HPXe gas-exchange MRI was performed at 1, 2, 3, and 4 months from the initial urethane administration (i.e., 0, 1, 2, and 3 months from commencement of EP treatment).

For the early-phase intervention group, prior to the administration of the urethane-saline solution, a 20 *μ*L solution of EP in saline was intratracheally administered to each mouse (2.6 mg/kg). Urethane administration was then performed as described above and thereafter, EP administration was continued on five consecutive days within one week for five months. HPXe gas exchange MRI was performed at 1, 2, 3, and 4 months from the initial urethane administration. After 5 months, mice of the late- and early-phase intervention groups were euthanized with a lethal dose of carbon dioxide gas to measure the number and size of lung surface tumors. Again, the survival rates of the whole 5-month procedure were 100% for the EP-treated group, after which mice were euthanized.

### 2.2. ^129^Xe Polarization and Gas Supply

HPXe was produced in a similar manner as previously described [9]. In brief, a gas mixture of 70% Xe (natural abundance, 26% ^129^Xe) and 30% N_2_ was supplied to a home-built continuous-flow ^129^Xe polarizer from a pre-mixed cylinder (Japan Air Gases Ltd., Tokyo, Japan) at a pressure of 0.15 atmospheres. The gas mixture was flowed continuously through a glass cell and ^129^Xe was polarized to ∼10% by irradiating a volume Bragg grating external-cavity diode laser with an output of 171 W and a wavelength of 794.6 nm (AW-SEOP; Aurea Works Corporation) onto the glass cell (see [20]). HPXe was subsequently compressed to atmospheric pressure via a diaphragm pump (LABOPORT® N86 KN.18, KNF Neuberger GmbH, Freiburg, Germany) and delivered directly and continuously from the polarizing cell to the mouse in the magnet. The gas mixture was flowed continuously at a rate of 50 mL/min to each mouse and was mixed with O_2_ (continuously supplied at 9 mL/min) and N_2_ (as a balance) in the mouse mask. The percentages of Xe, N_2_, and O_2_ spontaneously inhaled by the mice were 59%, 26%, and 15%, respectively.

### 2.3. MRI

All MRI measurements were performed on an Agilent Unity INOVA 400WB NMR spectrometer system (Agilent Technologies, Inc., Santa. Clara, CA, USA). A 9.4T vertical magnet with a bore width of 89 mm (Oxford Instruments Plc., Oxford, UK) was used. A self-shielded gradient probe was used in combination with Litz volume RF coils of 34 mm inner diameter, tunable to the Larmor frequencies of ^129^Xe (110.6 MHz) and ^1^H (399.6 MHz) (Clear Bore DSI-1117, Doty Scientific, Inc., Columbia, SC, USA). To acquire respiratory-gated images from spontaneously breathing mice, a respiratory sensor was used to synchronize the acquisition of HPXe lung images with inspiratory or expiratory phases as described previously [9].

HPXe images were acquired using a balanced steady-state free precession (bSSFP) sequence [9, 10] with either four 180° pulses or two 90° pulses to extract information about pulmonary gas exchange or ventilation function, respectively. Acquisition parameters are as follows: 1000-*μ*s Gaussian-shaped RF pulse of flip angle *α* = 40°; acquisition bandwidth, 88 kHz; TR/TE = 3.6 ms/1.8 ms; echo train length, 8; number of shots, 4; number of averages, 8; coronal slice thickness, 20 mm; matrix, 64 × 32 with a field of view of 80 × 25 mm^2^. Acquisition was commenced after confirming a steady state signal by monitoring ^129^Xe MR spectra obtained by the application of an 8° hard RF pulse with an interval of two seconds.

### 2.4. Assessment of Pulmonary Function

Parameters of pulmonary gas exchange, *f*_*D*_ (%), and fractional ventilation, *r*_*a*_, were derived from HPXe MR images as described previously [9]. Here, *f*_*D*_ is the rate of HPXe magnetization diffusion from the gaseous Xe (in the alveoli) to Xe in the dissolved-phase (lung tissue and blood) within a given exchange time, as evaluated by the xenon polarization transfer contrast (XTC) method [21]. The XTC image was generated by acquiring bSSFP gas ventilation images at expiration, separated by the application of four frequency-selective 180° inversion pulses (interpulse delay 20 ms) at the Larmor frequency of dissolved-phase ^129^Xe. The resulting ventilation image intensities were then compared with those acquired without irradiating inversion pulses.

The fractional ventilation, *r*_*a*_, is the alveolar volume fraction of gas turned over in a single breath. After gas-phase HPXe magnetization in the lung was destroyed by two saturation 90° prepulses, a set of inspiratory images was acquired after *n* breathing cycles. *n* was incremented from 1 to 10 in steps of one, and then to 12, 15, and 20. From these images, a *r*_*a*_ map was obtained by linear least squares fitting of the signal intensity as a function of *n*.

Maps of *f*_*D*_ and *r*_*a*_ were calculated pixel-by-pixel using in-house MATLAB (MathWorks, Inc., Natick, MA, USA) routines. Maps were subsequently averaged to obtain whole lung *r*_*a*_ and *f*_*D*_ values; and these values were compared between groups.

### 2.5. Lung Tumor Count

After HPXe MRI, mice were euthanized and lungs were extracted and immersed in 10% formalin at 25 cmH_2_O for at least 1 week. Surface tumors were counted, and tumor diameter was measured using calipers.

### 2.6. Histology

After lung tumor counting, the lungs of NC and LC model mice were processed for histology by staining with hematoxylin and eosin (H&E). Four coronal H&E-stained whole lung slices were obtained from each mouse and then captured using a digital microscope (Celestron LCD Microscope PRO <CE44345>; Celestron, LLC., Torrance, CA, USA).

In order to monitor morphological progression of the lung cancer model by urethane administration, three additional groups of LC model mice (“histological analysis” groups) were prepared at 1, 2, and 3 months after urethane injection (*N* = 3 mice for each timepoint) and histological images were obtained using the same protocol as described above.

### 2.7. Statistical Analysis

Statistical analysis was performed by Student's *t*-test or one-way ANOVA with Tukey–Kramer post-hoc analysis to identify significant differences between the NC, LC model, and EP-treated groups. All data are presented as mean ± standard deviation and/or box-and-whisker plots, and differences in functional parameters are considered significant at the *P* < 0.05 level.

## 3. Results

**Figure 2:**
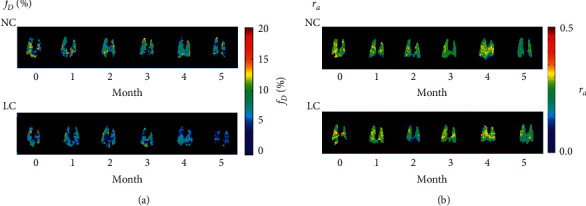
Representative parametric maps of longitudinal changes in (a) *f_D_* and (b) *r_a_* derived from mice in the negative-control (NC) group (*n* = 5) and lung cancer (LC) model group (*n* = 5), respectively.

**Figure 3:**
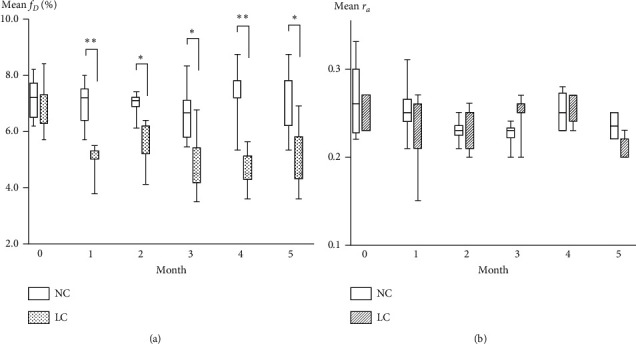
Box plots of the temporal change of mean (a) *f_D_* and (b) *r_a_* values for all mice, separated by group, as a function of time postinjection of saline (NC) or urethane (LC). Significant differences between groups are indicated by solid lines (^*∗*^: *P* < 0.05, ^*∗∗*^*P* < 0.01).

Figures 2 and 3 show longitudinal *f*_*D*_ (gas exchange) and *r*_*a*_ (ventilation) maps and box plots of average values, respectively, over the 5-month measurement period, obtained from the NC and LC model mice groups. The average *f*_*D*_ value of the LC model mice was significantly reduced compared with that of the NC mice at 1-month after urethane injection (*f*_*D*_NC_1m_ = 7.0 ± 0.9% versus *f*_*D*_LC_1m_ = 5.0 ± 0.7%, *P* < 0.01). Throughout the remaining observation period of 5 months, the *f*_*D*_ of the LC model mice continued to be significantly lower than that of the NC mice. In stark contrast, NC and LC mean *r*_*a*_ values were not statistically different over the measurement period, and neither group showed tendency for decline in ventilation function. The spatial distribution of *r*_*a*_ was relatively uniform compared with that of *f*_*D*_, which exhibited considerable spatial heterogeneity, particularly in LC model mice.

**Figure 4:**
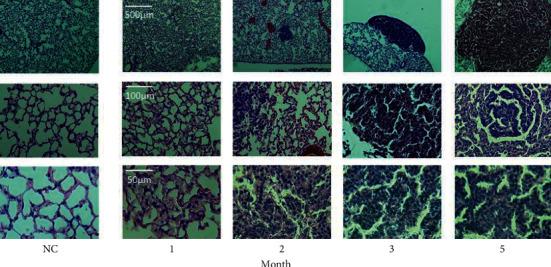
Representative examples of microphotographs of H&E-stained histology slides obtained from the LC model mice at 1, 2, 3, and 5 months post-urethane administration (right). For comparison, microphotographs of a representative NC mouse obtained at 5 months are also shown on the left.

**Figure 5:**
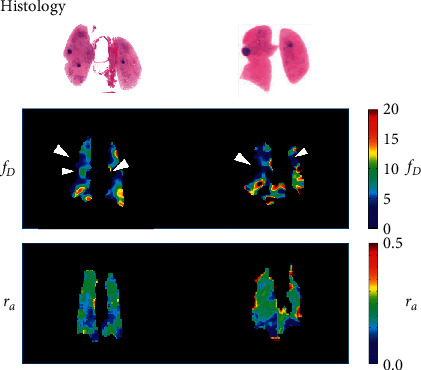
Qualitative spatial comparison of the HPXe MRI parametric maps (*f_D_* and *r_a_*) and histology slides obtained from two examples of LC model mice. Arrows indicate deficits in *f_D_* that correspond spatially with the location of tumors on histology.

Figure 4 shows representative histological microphotographs as a function of scan timepoint for the histological analysis (LC) subgroup, and at 5 months for the NC group. Precancerous lesions classified as alveolar epithelial hyperplasia were observed at 1 month after urethane injection [22]. This was followed by a mixture of alveolar epithelial hyperplasia and atypical adenomatous hyperplasia at 2 months, and adenoma at 3 and 5 months.  Figure 5 shows a qualitative visual comparison between the *f*_*D*_ and *r*_*a*_ maps and histological images for two representative LC model mice at 5 months. Regions of low *f*_*D*_ exhibited good spatial correspondence with tumor regions on histological images.

**Figure 6:**
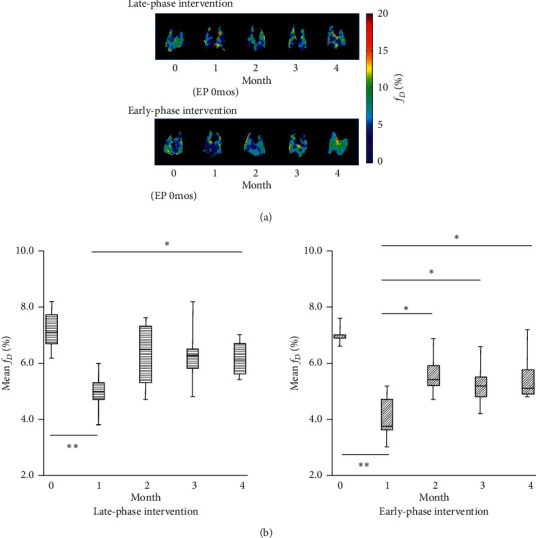
(a) Representative parametric maps of longitudinal changes in *f_D_* derived from EP-treated mice in the late- and early-phase intervention groups. (b) Box plots of the temporal change of mean *f_D_* values for EP-treated mice, separated by late- and early-phase intervention groups, as a function of time post-injection of urethane. Significant differences between groups are indicated by solid lines (^*∗*^*P* < 0.05, ^*∗∗*^*P* < 0.01).

Figures 6(a) and 6(b) show longitudinal *f*_*D*_ maps and average values, respectively, over the 4-month measurement period, obtained from the EP-treated groups. For both of the late- and early-phase intervention groups, the average *f*_*D*_ value at 1 month after urethane injection was significantly decreased compared with that of the base line (0 months), in a similar manner to that observed in the LC group. For the late-phase intervention group, the average *f*_*D*_ value significantly increased at 4 months after urethane injection (i.e., 3 months after commencing EP treatment) when compared with that at 1 month. In contrast, for the early-phase intervention group, the average *f*_*D*_ value was significantly improved (higher) at 2 months after urethane injection (2 months after commencing EP treatment) and continued to be higher for the remaining observation period when compared with that at 1 month.

**Figure 7:**
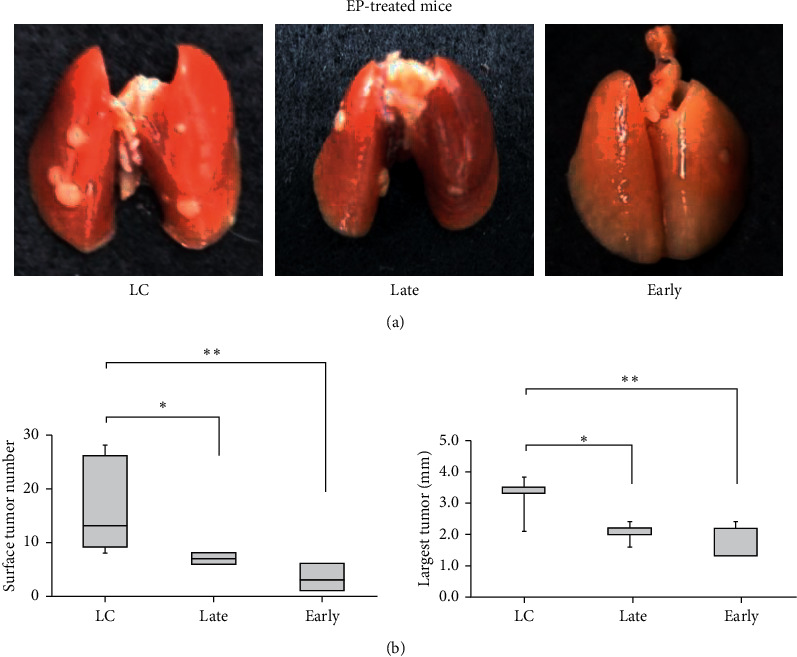
(a) Representative photographs of lungs obtained from LC and EP-treated mice. (b) Box plots of the number of solid tumors and the diameter of the largest tumor of LC and EP-treated mice. Late and early: late- and early-phase intervention groups.

Figure 7(a) shows representative photographs of the lungs of the LC, late-, and early-phase EP-intervention groups, highlighting several surface tumors in the LC group and a decreased number for the intervention groups.  Figure 7(b) shows the total tumor number and size of the largest tumor obtained from the LC, late-phase intervention, and early-phase EP-intervention groups. The median tumor numbers of the late-phase and early-phase intervention groups were 7 and 3, respectively, and significantly decreased compared with that of the LC mice (median: 13). The median size of the largest tumor of the late-phase (2.2 mm) and early-phase (2.2 mm) intervention groups was also significantly lower than that of the LC mice (3.5 mm). There was no significant difference between the late- and early-phase intervention groups.

## 4. Discussion

In this study, lung functional assessment by HPXe MRI revealed abnormalities in gas-exchange rate, *f*_*D*_, in a urethane model of lung cancer in mice. This was characterized by a significantly lower mean *f*_*D*_ value compared with that of the NC group at 1 month after urethane injection (Figures 2 and 3) and consistent gas exchange impairment for the remainder of the 5-month observation period. In contrast, the *r*_*a*_ of the LC model mice was not significantly different to that of the NC mice, despite the fact that solid tumor lesions were confirmed histologically at 3 months after urethane administration. However, since we acquired “projection” images with no anterior-posterior spatial selectivity, the images represent the average gas distribution in that direction, reducing the sensitivity to regionally localized ventilation abnormalities. While tumors are known to be associated with ventilation defects on HPXe lung images, these defects are typically locally confined to cancer regions and are not expected to affect large regions of the mice lung [11]. Since the scale of tumors in this study is relatively small (see Figures 5 and 7), this likely prevents the observation of neoplastic lesions on *r*_*a*_ maps. As such, we postulate that *r*_*a*_ may be an unsuitable parameter for study of this cancer model, and any observed ventilation abnormality may reflect accompanying respiratory disorders, rather than the tumors themselves. For similar spatial selectivity reasons, it was difficult to locally identify the tumor regions on the *f*_*D*_ maps; however, as shown in Figure 5, gas exchange defects were observed from two mice to be spatially correspondent to the solid tumor regions, which likely predominantly reflects true gas exchange pathology.

To elucidate the relationship between pulmonary gas exchange function and morphology during cancer progression, histological analysis was performed on subgroups of mice at 1, 2, and 3 months after urethane injection (Figure 4) and a range of neoplastic lesions was observed, similar to that reported in [23]. Alveolar epithelial hyperplasia was observed at 1 month after urethane injection, while adenoma was observed after 3 months after urethane injection. Therefore, the decrease of *f*_*D*_ observed at 1 month in LC model mice (Figures 2(a) and 3(a)) can be associated with alveolar epithelial hyperplasia. To the best of our knowledge, this is the first report of the application of HPXe MRI to study gas exchange abnormalities in alveolar epithelial hyperplasia.

The reduction in *f*_*D*_ appears to correspond to the thickening of alveolar epithelium, similar to the reported result that *f*_*D*_ in a mouse model of lung fibrosis was also reduced due to thickening of the alveolar epithelium [9]. In the XTC measurement performed to derive *f*_*D*_, HPXe magnetization in the dissolved-phase is inverted, and this inverted HPXe magnetization is retransferred to the gas-phase, which causes a decrease of the gas phase signal that is used to infer an *f*_*D*_ map. In our experiments, we performed XTC by applying 4 inversion pulses with interpulse delay of 20 ms, i.e., 80 ms of exchange time. When the alveolar epithelium becomes thickened, that is, the volume of the dissolved-phase compartment increases, it may be expected that *f*_*D*_ would also increase; however this is contrary to our finding of reduced *f*_*D*_ in LC mice. We postulate that HPXe might not reach equilibrium between the gas- and dissolved-phase compartments within 80 ms in thickened epithelia, and as a result, some of the inverted HPXe magnetization might not return to the gas-phase within that time. Since the inverted magnetization remaining in this dissolved-phase does not affect the gas-phase signal, the *f*_*D*_ of the LC model mice might decrease. In our previous report using healthy mice, it took about 50 ms for the saturated dissolved-phase HPXe magnetization to reach equilibrium with that of the gas-phase [24]. Therefore, when the volume of the dissolved-phase compartment is increased due to alveolar hyperplasia, the time required to reach the equilibrium is likely extended.

According to [25], in lung cancer model mice, inflammation can occur as early as one week after urethane administration, which is reported to be correlated with the development and progression of lung cancer. Previously, we reported *f*_*D*_ reductions in mice models of COPD and lung fibrosis [9, 10], attributed to bronchial and alveolar wall thickening resulting from inflammation. Therefore, in the present study, the alveolar epithelial hyperplasia observed histologically at 1 month after urethane injection was likely accompanied by the pulmonary inflammation. Accordingly, the observed impairment of gas exchange (reduction in *f*_*D*_) was possibly caused by pulmonary inflammation associated with alveolar epithelial hyperplasia, preceding the appearance of neoplastic lesions.

The major mechanism of alveolar epithelial hyperplasia is considered to be damage of the alveolar epithelium caused by pulmonary inflammation as mentioned above [25, 26]. This inflammation results in continuous release of inflammatory cytokines from inflammatory cells involving High-Mobility-Group-Box1 (HMGB1), which is one of the damage-associated-molecular-pattern (DAMP) molecules. In our previous reports, we suggested that HMGB1 was associated with *f*_*D*_ reductions in mice models of COPD and lung fibrosis due to alveolar structural changes caused by inflammation [9, 10]. HMGB1 has been implicated not only in various inflammatory diseases but also in cancer development [27], and overexpression of HMGB1 has been observed in non-small cell lung cancer patients [28]. Once HMGB1 released from necrotic or apoptotic cells into the extracellular space binds to advanced glycation end product receptors and toll-like receptors in lung precancerous lesions, it activates the transcription factor nuclear factor-kappa B (NF-*κ*B) signaling pathways and exacerbates inflammation and pathology [27].

EP has been shown to exhibit anti-inflammatory effects by downregulating HMGB1 expression through inactivation of NF-*κ*B [29, 30]. Based on this mechanism, EP has been reported to show anticancer effects *in vivo* on liver cancer [31–33], mesothelioma [34], diffuse large B-cell lymphoma [35], and angiogenesis using Lewis lung carcinoma cells subcutaneously injected into the flanks of mice [36]. Therefore, in order to test the hypothesis that the *fD* reduction observed at 1 month after urethane administration here is due to inflammation associated with HMGB1 secretion, we examined the therapeutic effect of EP on LC model mice by HPXe MRI. In the late-phase intervention group, *fD* gradually increased and showed a significantly larger value at 4 months than that at 1 month (Figure 6). In addition, the number and size of surface tumors in the late-phase intervention group were significantly decreased compared with those of the LC mice (Figure 7).

For the early-phase intervention group, even though EP was administered prior to urethane administration, the *f*_*D*_ at 1 month was significantly lower than that of the baseline (0 months) to a similar degree as the LC group and late-phase intervention group. However, following that, the *f*_*D*_ value recovered to a significantly larger value than that of the baseline at 2 months post urethane administration. This recovery in *f*_*D*_ was maintained for the remaining observation period (Figure 6). The number of tumors in the early-phase intervention group was significantly reduced to about 25% of that of the LC group, and the median size of the largest tumor was also significantly reduced (Figure 7). Combining all results from the two intervention groups, the hypothesis of EP action described above appears to be supported.

## 5. Limitations

One of the main limitations of this study is that direct imaging of the dissolved-phase was not performed. Accordingly, it is challenging to explain the exact mechanisms of the decrease in gas exchange function due to inflammation in the LC model mice. Direct simultaneous imaging of the dissolve-phase and gas-phase HPXe would help to further clarify our hypothesis that the diffusion of HPXe between the gas- and dissolved-phase compartments does not reach equilibrium in 80 ms of exchange time as mentioned above [17].

A further limitation of this study is that morphological evaluation of the lung cancer was difficult. Hitherto, the morphological evaluation of urethane-induced lung cancer mouse models has been performed by x-ray CT, MRI, and PET [25, 37–42]. According to those reports, the presence of cancer was confirmed 2 months after urethane administration by CT [25, 37, 38]. The evaluation of efficacy of drugs for lung cancer was also attempted by CT, and the effects of anti-inflammatory drugs on inhibition of tumor growth have been observed [43, 44]. In addition, the observation of lung cancer has been shown to be feasible by ^1^H MRI and PET, and its longitudinal changes were also evaluated [39–42]. However, these reports were based on observations after cancer nodules had already developed, which is at a later stage than our functional evaluation. Therefore, the methods presented in the present study may be used in a complementary means with other imaging modalities to evaluate the efficacy of drugs after starting treatment.

## 6. Conclusions

In a murine model of lung cancer induced by urethane injection, lung functional impairment during cancer progression was detected by the HPXe MRI metric of gas-exchange, *f*_*D*_, concordant with the onset of alveolar epithelial hyperplasia. This metric shows potential as an indicator of tumor microenvironment, including inflammatory processes, preceding the appearance of atypical adenomatous hyperplasia and adenoma. EP treatment was found to improve the lung cancer induced gas exchange impairment and inhibit further lung cancer development. These findings suggest that HPXe dissolved-phase MRI is suitable for longitudinal monitoring and assessment of treatment response in preclinical lung cancer models.

## Data Availability

The data used to support the findings of this study are available from the corresponding author upon request.

## Abbreviations

LC: Lung cancer
NC: Negative control
EP: Ethyl pyruvate
HP: Hyperpolarized.
